# Exposure to multisensory and visual static or moving stimuli enhances processing of nonoptimal visual rhythms

**DOI:** 10.3758/s13414-022-02569-1

**Published:** 2022-10-14

**Authors:** Ourania Tachmatzidou, Nadia Paraskevoudi, Argiro Vatakis

**Affiliations:** 1 Department of History and Philosophy of Science, National and Kapodistrian University of Athens, Athens, Greece; 2 Multisensory and Temporal Processing Lab (MultiTimeLab), Department of Psychology, Panteion University of Social and Political Sciences, 136 Syngrou Ave, 17671 Athens, Greece

**Keywords:** Rhythm, Multisensory perception, Motion, Perceptual learning, Audiovisual

## Abstract

Research has shown that visual moving and multisensory stimuli can efficiently mediate rhythmic information. It is possible, therefore, that the previously reported auditory dominance in rhythm perception is due to the use of nonoptimal visual stimuli. Yet it remains unknown whether exposure to multisensory or visual-moving rhythms would benefit the processing of rhythms consisting of nonoptimal static visual stimuli. Using a perceptual learning paradigm, we tested whether the visual component of the multisensory training pair can affect processing of metric simple two integer-ratio nonoptimal visual rhythms. Participants were trained with static (AVstat), moving-inanimate (AVinan), or moving-animate (AVan) visual stimuli along with auditory tones and a regular beat. In the pre- and posttraining tasks, participants responded whether two static-visual rhythms differed or not. Results showed improved posttraining performance for all training groups irrespective of the type of visual stimulation. To assess whether this benefit was auditory driven, we introduced visual-only training with a moving or static stimulus and a regular beat (Vinan). Comparisons between Vinan and Vstat showed that, even in the absence of auditory information, training with visual-only moving or static stimuli resulted in an enhanced posttraining performance. Overall, our findings suggest that audiovisual and visual static or moving training can benefit processing of nonoptimal visual rhythms.

## Introduction

Rhythm perception is considered by most as tightly associated with the auditory system (e.g., Grahn, 2012; Grahn et al., 2011; Grondin & McAuley, 2009). This seems counterintuitive given that most everyday activities that require efficient processing of temporally structured patterns are inherently multisensory (Ghazanfar, 2013; Grahn & Brett, 2009; Su & Pöppel, 2012). Consider, for example, the rhythmic information contained in the dancing or walking act. We dance by synchronizing our movements to the music and our partner or we maintain rhythmic gait by integrating visual, auditory, tactile, and proprioceptive feedback from the environment. To date, however, most studies on rhythmic processing have focused primarily on auditory rhythms, thereby largely ignoring the contribution of the other senses to rhythm perception.

Recently, a small number of studies have started to investigate the intramodal, as well as the crossmodal differences in rhythm perception and discrimination (Grahn, 2012; Grahn et al., 2011; Hove et al., 2013). Intramodal studies in the auditory domain have shown that the presence of a periodic beat that yields salient physical accents and gives rise to a clear metrical structure enhances auditory rhythm processing as compared with rhythms with irregular temporal structure (Grahn, 2012; Phillips-Silver & Trainor, 2007). Indeed, the beneficial impact of “hearing the beat” of a rhythm (i.e., the regular pulse that serves as a temporal anchor around which events are organized; Iversen et al., 2009) facilitates rhythm processing and encoding (Grahn, 2012; Su, 2014b), as well as motor synchronization (Gan et al., 2015; Grahn, 2012; Grahn & Brett, 2007). Crossmodal studies have also reported modality-dependent effects on rhythm processing. Research has consistently shown an auditory advantage in rhythm perception, which has been attributed to the more fine-grained temporal resolution of the auditory as compared with the visual system (Collier & Logan, 2000; Grahn, 2012; Grahn et al., 2011; Patel et al., 2005). For example, a periodic rhythm can be efficiently processed by the auditory channel, while the same rhythm cannot be easily recognized when presented in the visual modality (Collier & Logan, 2000; Grahn et al., 2011; Patel et al., 2005). This is further supported by neuroimaging data that have demonstrated increased activity of timing-related areas (i.e., basal ganglia, putamen) when processing an auditory rhythm as compared with a rhythm mediated by static visual flashes (Grahn et al., 2011; Hove et al., 2013).

Recent data, however, have challenged the currently supported visual inferiority in rhythm processing by demonstrating that rhythm discrimination performance is contingent upon the reliability of the stimulus presented (Gan et al., 2015; Grahn, 2012; Hove et al., 2013). Specifically, it has been suggested that the auditory dominance in rhythm processing may be partly due to the use of nonoptimal visual stimuli such as static flashes (Barakat et al., 2015) that lack spatiotemporal information, while motion—a more optimal visual stimulus (e.g., Ernst & Banks, 2002; Welch & Warren, 1980)—has been found to increase the temporal reliability of visual rhythm encoding (Gan et al., 2015; Grahn, 2012; Hove et al., 2013). The optimality of visual moving stimuli in rhythm perception was first investigated by Grahn (2012). Specifically, she directly compared auditory rhythms with visual rhythms with the latter being formed by a moving line. Three types of rhythmic patterns were used: (a) metric simple (i.e., integer-ratio rhythms with regular temporal accents that provide a clear metrical structure), (b) metric complex (i.e., integer-ratio rhythms with irregular temporal accents), and (c) nonmetric rhythms (i.e., non-integer-ratio rhythms with irregular temporal accents). In each trial, three rhythmic sequences were presented, and participants had to report whether the third sequence differed from the other two or not. The results showed higher accuracy for auditory trials as compared with visual ones, thus supporting the auditory advantage in rhythm processing (Collier & Logan, 2000; Patel et al., 2005). However, performance in the visual trials was also significantly improved, but this was only found for the metric simple rhythms and not the metric complex and nonmetric rhythms, indicating that visual rhythm encoding requires a clear metrical structure. Although these findings support the auditory advantage in rhythm processing, they also demonstrate that visual rhythm processing can also be enhanced when moving stimulation is used.

In addition to the beneficial impact of visual moving stimuli on rhythm perception, multisensory stimulation with visual components consisting of biological movement have also been found to affect the encoding and processing of rhythmic patterns (Su, 2014a, 2014b, 2016; Su & Salazar-López, 2016). Studies using point-light human figures along with auditory rhythmic patterns (Su, 2014a, 2014b, 2016; Su & Salazar-López, 2016) have shown improved discrimination accuracy for audiovisual metric simple (Su, 2014b) and metric complex rhythms (Su, 2014a) as compared with auditory-only rhythms. This improvement is also in line with several studies reporting enhanced performance in multisensory as compared with unisensory trials (e.g., Alais & Cass, 2010; Roy et al., 2017; Shams et al., 2011). However, no study to-date has directly assessed whether animate and inanimate moving stimuli exert differential influences on rhythm discrimination given that two different mechanisms have been suggested to mediate temporal processing for these types of stimuli (Carrozzo et al., 2010).

Given the increasing evidence suggesting that certain types of visual (e.g., Grahn, 2012; Hove et al., 2013) and multisensory stimulation (e.g., Su, 2014a, 2014b) affect rhythm processing, exposure to such sensory rhythmic stimulation could potentially facilitate subsequent processing of visual rhythms. Facilitation of visual rhythms after training has recently been reported in a perceptual learning study by Barakat et al. (2015). Specifically, after receiving visual, auditory, or audiovisual training, participants in this study had to discriminate between two visual-only rhythmic sequences composed of visual empty intervals (i.e., demarcated by static flashes occurring at the onset and offset of each interval). Results showed that visual training did not contribute to an enhanced posttraining performance, while both the auditory and multisensory training groups were significantly better during the posttraining session. More importantly, these latter two groups did not differ in their posttraining performance, suggesting that multisensory training did not enhance rhythm perception more than the auditory training. One could, thus, argue that the posttraining enhancement observed was auditory-driven, while it remains unanswered whether the absence of posttraining improvement for the visual training group was due to the use of nonoptimal static stimuli (Grahn, 2012; Hove et al., 2013).

As far as we know, no study has yet examined the effects of modality and stimulus attributes such as visual movement or animacy on enhancing rhythm perception in a task consisting of static stimuli. Additionally, no attempts have been made to manipulate the animacy of the training stimulus and directly compare performance following exposure to animate and inanimate movement so as to assess whether the former benefits rhythm processing more than the latter. To address this gap, we examined whether multisensory training with different types of visual stimulation (i.e., static vs. moving and inanimate vs. animate) yield differential learning effects in a subsequent visual rhythm discrimination task consisting of static visual stimuli. We hypothesized that training with audiovisual rhythms, particularly those containing visual movement, would improve the processing of visual static rhythmic patterns due to the visual system's high spatial resolution and enhanced motion processing (e.g., Hove et al., 2013; Welch & Warren, 1980). Furthermore, we reasoned that if biological motion has a beneficial impact on rhythm processing (Su, 2014a, 2014b), then training with auditory rhythms accompanied by animate visual movement would yield better discrimination performance in a subsequent visual-only rhythm discrimination task as compared with training with moving, yet inanimate, visual stimuli.

## Experiment 1

### Methods

#### Participants

Fifty-three university students (47 female) aged between 19 and 48 years (mean age = 24 years) took part in the experiment. All participants reported having normal or corrected-to-normal vision and normal hearing. All were naïve as to the purpose of the experiment. The experiment was performed in accordance with the ethical standards laid down in the 2013 Declaration of Helsinki and informed consent was obtained from all participants. To control for potential confounding factors, participants with extensive (over 5 years) musical and/or dance training were removed from further analysis (cf. Grahn & Rowe, 2009; Iannarilli et al., 2013).

#### Apparatus and stimuli

The experiment was conducted in a dimly lit and quiet room. The visual stimuli were presented on a CRT monitor with 60 Hz refresh rate, while the auditory stimuli were presented using two loudspeakers (Creative Inspire 265), placed to the left and right of the monitor. The experiment was programmed using OpenSesame (Version 3.1; Mathôt et al., 2011).

Three types of visual stimuli were utilized to create the visual stream of the audiovisual rhythmic sequences: (a) red and green static circles, (b) a moving bar, and (c) a human point-light figure (PLF). Both the static circles and the moving bar were created using Adobe Illustrator CS6. The moving bar was designed with six different orientations, each one pointing to a different position (separated by approximately 30°) around a central axis of rotation, so that apparent movement could be induced when presented sequentially (cf. Grahn, 2012). The PLF was adopted from the Atkinson et al.’s (2004) stimulus set and was processed in Adobe Premiere Pro CS5. The PLF moved vertically, starting from an upright position, then bending down and, finally, returning to its initial position. We used this kind of movement, since vertical human body movements have been suggested to mediate rhythm more efficiently than horizontal body movements (Nesti et al., 2014; Toiviainen et al., 2010). All stimuli can be accessed online (https://osf.io/pzc2t/).

The auditory stream of the rhythmic sequences utilized was created using Audacity and was composed of two types: (a) a sinewave tone (sampling frequency 44110 Hz) of 43 ms in duration and (b) a pink noise (sampling frequency 44110 Hz) of 50 ms in duration. The former sound was used to create the auditory rhythmic patterns, while the latter the beat sequences. Both the auditory tones and the beat stimuli were presented at 76 dB (as measured from the participant's ear position).

#### Design

**Fig. 1:**
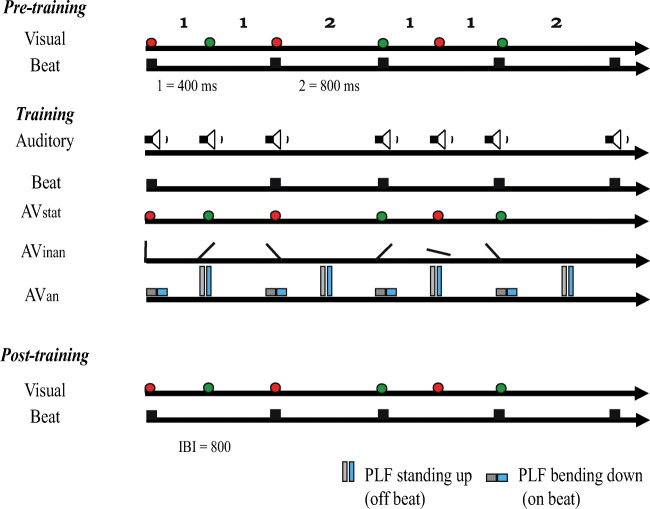
Schematic of the design and the stimuli used for the rhythmic patterns in Experiment 1. The rhythmic pattern shown here is for the 6-interval 112112 rhythm with 1 = 400 ms and 2 = 800 ms. All participants initially performed a pretraining session consisting of static circles (red and green ellipses) and a regular beat occurring every 800 ms (black square). They were, subsequently, randomly assigned to one of the three training groups and received two training sessions that were separated by a day. The first group (AVstat) was trained with auditory tones and static circles of changing colours (red and green ellipses). The second group (AVinan) was presented with auditory tones and a bar “moving” to different screen locations (black line). The third group (AVan) was trained with auditory tones and a human point-light figure starting in an upright position (grey-blue lines) and then bending down (grey-blue squares). After training, all participants completed the final posttraining session that was the same as the pretraining. (Colour figure online)

The experiment took place in two separate days (24 to 48 hours apart), with the experimentation of each day lasting approximately 50 minutes. The experiment consisted of four sessions: a) a pretraining session, b) two training sessions, and c) a posttraining session (cf. Barakat et al., 2015; see Fig. 1). For all sessions, the participants completed a two alternative forced choice (2AFC) rhythm discrimination task (i.e., 'same' or 'different'). In each trial, a fixation point was initially presented for 1000 ms followed by the first rhythmic pattern (i.e., “standard”). After 1,100 ms (interstimulus interval; ISI), the second rhythmic sequence (i.e., “comparison”) was presented and participants provided a self-paced response. The intertrial interval (ITI) was set at 1,200 ms.

The rhythmic sequences used were metric simple rhythms (cf. Grahn, 2012; Grahn & Brett, 2007) that consisted of six elements of either a short (400 ms) or a long interval (800 ms). The intervals were, thus, related by integer ratios, where 1 = 400 ms and 2 = 800 ms, and had a regular grouping with the beat occurring regularly every two units (cf. Drake, 1993)—that is, every 800 ms (interbeat interval, IBI; cf. Grahn & Brett, 2007). Five rhythmic sequences were used as ‘standard’ and ‘comparison’ intervals (i.e., Rhythm A: 111122, B: 112112, C: 112211, D: 211211, and E: 221111), resulting in a factorial 5 × 5 design with 25 rhythm pairs in total (i.e., AA, AB, AC, AD, AE, BA, BB, BC, BD, BE, CA, CB, CC, CD, CE, DA, DB, DC, DD, DE, EA, EB, EC, ED, and EE).

On Day 1 of the experiment, participants completed a set of five practice trials to familiarize themselves with the task. Subsequently, they all performed the pretraining session and the first training session. On Day 2, participants started with the second training session that was followed by the posttraining test (that was identical to the pretraining test).

The pre- and posttraining sessions were composed of visual-only rhythms that consisted of static circles of changing colours (see Fig. 1). During each trial, a circle appeared on the screen and lasted for the whole duration of the respective interval (i.e., 400 or 800 ms). Once the first interval ended, the circle changed colour (green or red, based on the previous circle), which represented the onset of the next element of the rhythmic sequence. Each one of the two rhythms in a given trial consisted of six elements (i.e., six circles). The pre- and posttraining sessions consisted of four repetitions of each rhythm pair, resulting in 100 trials per session in total. Each session lasted approximately 30 minutes.

For the training phase, all participants were randomly assigned to one of three training groups: audiovisual static circles (AVstat), moving bar (i.e., inanimate stimulus; AVinan), or PLF (i.e., animate stimulus; AVan) group. During the training sessions, participants received feedback for their responses. Each training session included 3 repetitions of each rhythm pair (i.e., 75 trials per session in total) and lasted approximately 20 minutes. The auditory stimulation was the same across the three training groups, with the auditory tones occurring at the onset and offset of each interval, and the beat being presented every 800 ms. The first group (AVstat; *N* =16, 15 female, age range: 19–38, mean age = 25.2 years) was trained with rhythms consisting of auditory tones and static circles of changing colours. The presentation of the circles was the same as in the pre- and posttraining with the sole exception that, here, the onset of each circle was accompanied by an auditory tone. The second group (AVinan; *N* = 20, 15 female, age range: 19–48, mean age = 23.5 years) was trained with audiovisual rhythms consisting of a moving bar (cf. Grahn, 2012) that was accompanied by auditory tones. In this case, a line was initially presented in a vertical position and once the rhythmic pattern started, the line changed positions sequentially around a central axis of rotation. The third group (AVan; *N* = 15, 15 female, age range: 21–39, mean age = 21.9 years) was trained with a human PLF with each PLF cycle lasting 800 ms. Thus, the transition from the upright position to the lowest position of the PLF and the reverse lasted 400 ms each, that is, the beat always occurred at the lowest position of the PLF as suggested by previous studies (cf. Su, 2014a, 2014b).

#### Procedure

The participants received detailed verbal instructions prior to the start of the experiment, and they were allowed to ask for any clarification. Prior to the start of the experiment, participants completed a practice session to familiarize themselves with the task. They, subsequently, performed the pretest and the first training session (Day 1) that was followed by the second training session and the posttest in Day 2. Participants self-initiated each session. Once both sequences were presented, they were instructed to report as accurately as possible whether the two rhythms differed or not, by pressing the keys “m” and “z” of the keyboard, respectively. Participants were informed that during the training sessions response feedback would be provided, while this would not be the case for the pre- and posttest. Finally, all participants were allowed to take a break between the experimental sessions.

### Results and discussion

Two participants were removed from the analysis due to formal musical and dance training. For all the analyses, Bonferroni-corrected *t* tests (where *p* < .05 prior to correction) were used for all post hoc comparisons. When sphericity was violated, Greenhouse–Geisser correction was applied. The alpha level was set to 0.05 and the confidence interval to 95%. Moreover, 90% confidence intervals around eta partial square (Steiger, 2004) are reported to facilitate future researchers (Thompson, 2002) and to provide further information on the sufficiency of the sample size (Calin-Jageman, 2018).

#### Training data

**Fig. 2:**
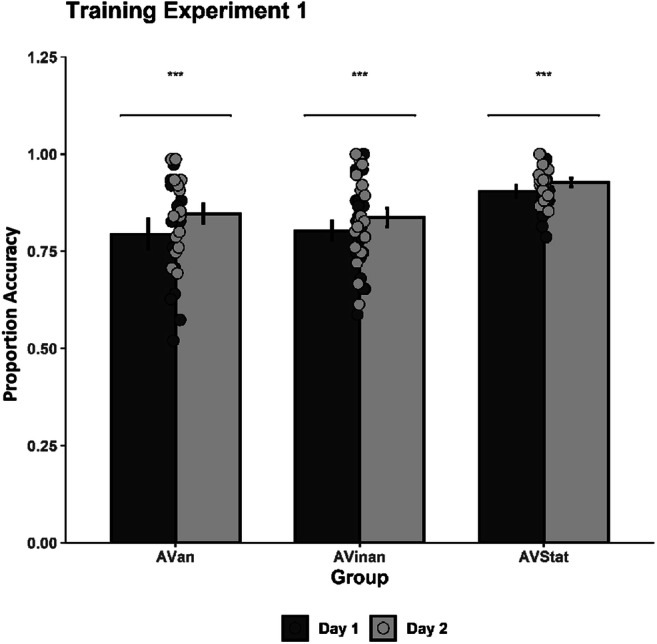
Mean discrimination accuracy during the two training sessions for the three training groups (AVstat, AVinan, AVan) in Experiment 1. Significant differences between the groups (*p* < .05) are indicated by the asterisk. The error bars represent the standard error of the mean

The training data (i.e., percentage correct detections of “same” or “different” rhythmic pairs) were analyzed via a mixed analysis of variance (ANOVA) with Training Session (2 levels: Session 1 vs. Session 2) and Rhythm Pair (25 levels) as the within-participant factors, and Group (3 levels: AVstat, AVinan, AVan) as the between-participants factor. The analysis showed a significant main effect of Group, *F*(2, 48) = 5.94, *p* = .005, η_p_^2^ = .20, CI [.04, .334], with the AVstat group performing significantly better (*M* = .916) as compared with both the AVinan (*M* = .819) and the AVan (*M* = .820) group (see Fig. 2). The higher performance of the AVstat group could be attributed to the prior exposure to the pretraining session (i.e., identical visual stimulation); however, it should be noted that both AVinan and AVan groups also reached high levels of performance accuracy with a mean accuracy over 80%. A significant main effect of Training Session was also obtained, *F*(1, 48) = 12.39, *p* < .001, η_p_^2^ = .21, CI [.058, .335], with all groups having higher accuracy scores during the second training session (*M* = .868) as compared with the first (*M* = .832). Thus, showing that even one training session was sufficient to yield higher discrimination accuracy for all three groups. We also obtained a significant main effect of Rhythm Pair, *F*(10.97, 526.55) = 11.61, *p* < .001, η_p_^2^ = .19, CI [.132, .228], with certain pairs having systematically lower accuracy (M_AA_ = .777, M_BB_ = .791, M_BC_ = .725, M_CB_ = .693, M_CC_ = .771, M_CD_ = .722, M_DD_ = .761, M_DE_ = .794) as compared with others that were significantly easier to discriminate (M_AC_ = .905, M_AD_ = .902, M_AE_ = .918, M_BA_ = .915, M_BE_ = .938, M_CA_ = .928, M_CE_ = .918, M_DA_ = .951, M_EA_ = .961, M_EB_ = .908). The data showed that the rhythms B, C, and D (i.e., 112112, 112211, and 211211, respectively) were particularly difficult to discriminate in certain types of pairing, yet the performance was still above chance level.

A significant interaction between Rhythm Pair and Group was obtained, *F*(21.94, 526.55) = 2.10, *p* = .003, η_p_^2^ = .08, CI [.014, .080], with the AVstat group having significantly higher accuracy scores in some rhythm pairs (M_AE_ = 1, M_BA_ = .969, M_CE_ = .990, M_DA_ = .990, M_DD_ = .885, M_EA_ = 1, M_EB_ = .979) as compared with the AVan (M_AE_ = .900, M_BA_ = .844, M_DA_ = .889, M_EA_ = .922, M_EB_ = .844) and AVinan (M_AE_ = .867, M_CE_ = .867, M_DD_ = .675) groups, while AVan performed significantly worse than the other two groups when the rhythm pair was BC (AVan = .500, AVinan = .875, AVstat = .750). Furthermore, a significant interaction between Training Sessions and Rhythm Pair was also obtained, *F*(13.56, 651.04) = 1.74, *p* = .046, η_p_^2^ = .04, CI [.002, .040]. The accuracy scores of numerous rhythm pairs were significantly lower during the first Training Session (M_AD_ = .837, M_AE_ = .856, M_BA_ = .869, M_CA_ = .876) in comparison to the second (M_AD_ = .967, M_AE_ = .980, M_BA_ = .961, M_CA_ = .980). The interactions between Training Session and Group, *F*(2, 48) = 0.63, *p* = .539, η_p_^2^ = .03, and Group, Training Session, and Rhythm Pair, *F*(27.13, 651.04) = 1.27, *p* = .162, η_p_^2^ = .05, did not reach significance.

#### Pre- and posttraining data

**Fig. 3:**
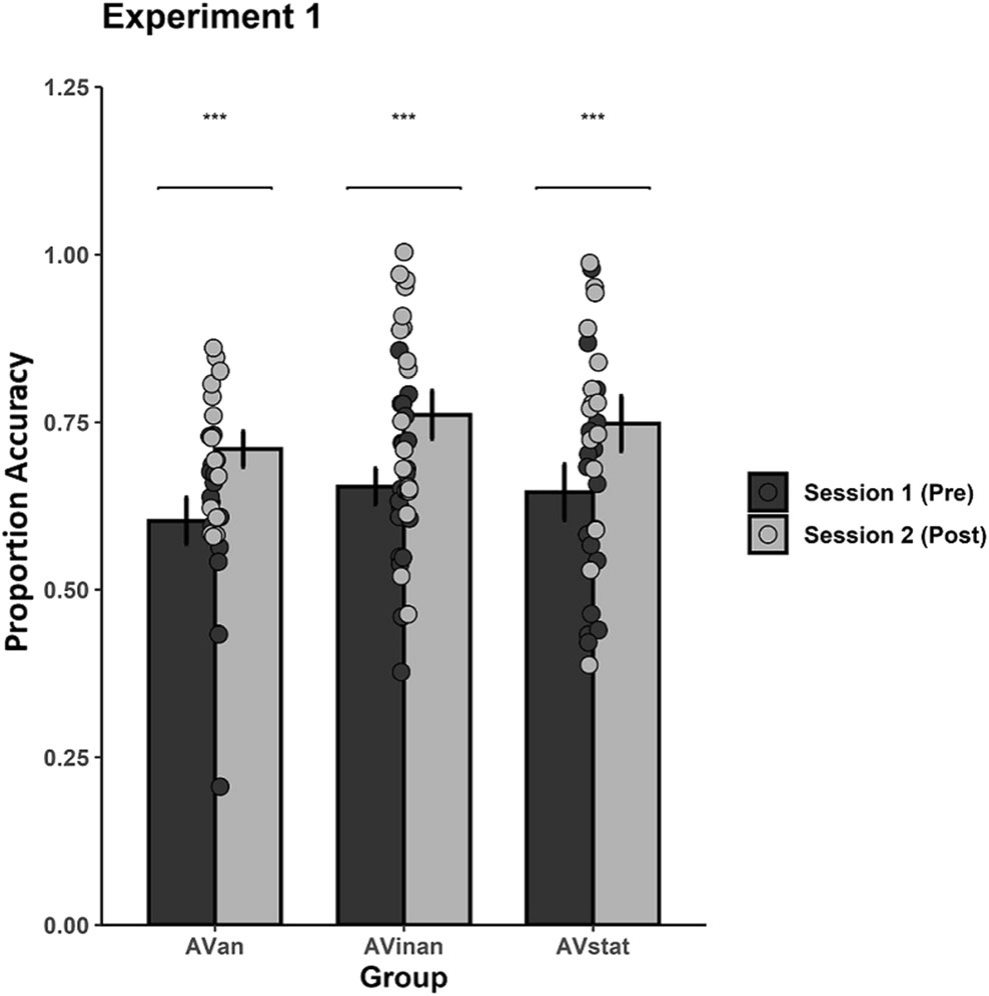
Mean discrimination accuracy during the two main sessions (pre- and posttraining) for the three training groups (AVstat, AVinan, AVan) in Experiment 1. Significant differences between the pre- and posttest sessions (*p* < .001) are indicated by two asterisks. The error bars represent the standard error of the mean

For the main analysis, we compared the pre- and posttraining performance to test for potential learning effects following training. The pre- and posttest responses were analyzed via a mixed ANOVA with Session (2 levels: Pretraining vs. Posttraining) and Rhythm Pair (25 levels) as within-participant factors, and Group (3 levels: AVstat, AVinan, AVan) as between-participants factor. A significant main effect of Session was obtained, *F*(1, 48) = 59.23, *p* < .001, η_p_^2^ = .55, CI [.383, .655], with all groups performing better (*M* = .742) during posttraining as compared with the pretraining session (*M* = .639; see Fig. 3). A significant main effect of Rhythm Pair was also obtained, *F*(13.44, 645.08) =13.21, *p* < .001, η_p_^2^ = .22, CI [.156, .245], with certain rhythm pairs being more accurately discriminated (i.e., M_AE_ = .819, M_EA_ = .833, M_EC_ = .821, M_EE_ = .836) as compared with others (i.e., M_BC_ = .537, M_CB_ = .480, M_CD_ = .615, M_DC_ = .620, M_DE_ = .561).

Further examination of this effect showed that Rhythm E (i.e., 221111) was easier to discriminate from other rhythms suggesting that this rhythmic pattern was more efficiently processed and maintained in memory as compared with the other rhythmic sequences. This was not the case for pairs including the Rhythms B (i.e., 112112), C (i.e., 112211), and D (i.e., 211211) that lead the participants to lower discrimination accuracy. No main effect of Group was obtained, *F*(2, 48) = 0.66, *p* = .521, η_p_^2^ = .03], while the interactions between Group and Session and between Group, Session, and Rhythm Pair did not reach significance, *F*(2, 48) = 0.01, *p* = .988, η_p_^2^ = .01, and *F*(26.98, 647.50) = 1.27, *p* = .162, η_p_^2^ = .05, respectively. These findings suggest that the type of the visual component presented during training did not modulate posttraining performance. However, we found a significant interaction between Group and Rhythm Pair, *F*(26.88, 645.08) = 1.68, *p* = 0.17, η_p_^2^ = .07, CI [.005, .058]. Specifically, although performance was equal between groups, the AVstat and AVinan groups differed significantly in their discrimination accuracy for the rhythm CC (*M* = .820 and .650, respectively). We also found a significant interaction between Session and Rhythm Pair, *F*(13.49, 647.50) = 4.46, *p* < .001, η_p_^2^ = .09, CI [.037, .102], with 18 out of 25 rhythm pairs being more accurately discriminated in the posttest as compared with the pretest. These findings demonstrate that training had a beneficial impact on discrimination performance for most rhythmic patterns.

Overall, the results of Experiment 1 showed that the different training stimulation utilized resulted in similar posttest performance for all training groups, despite the main effect of group during training. That is, irrespective of the training stimulus type (i.e., static or moving, animate or inanimate), the multisensory perceptual training implemented enhanced the processing of subsequently presented visual-only, static rhythms. The absence of group differences could be attributed to the fact that the auditory stimulation was identical for all training groups. Thus, it could be the case that the benefit obtained after training was driven solely or mainly due to the contribution of audition, thereby providing support for the modality appropriateness hypothesis (i.e., theory supporting that the most reliable modality will dominate the final percept depending on the task utilized; Welch & Warren, 1980). An alternative explanation of our findings could be the ease of the task. The results showed that, even during the pretraining session, most participants exhibited high discrimination accuracy, which could be due to low task difficulty. This ease of rhythm discrimination could be attributed either to the presence of the explicit beat (cf. Su, 2014a), the low complexity of the rhythms presented (that consisted of only two interval types; i.e., 400 and 800 ms; cf. Barakat et al., 2015; Drake, 1993), or the rhythm type utilized that had a clear metrical structure (i.e., metric simple rhythms; cf. Grahn, 2012; Su, 2014b).

The potential contribution of audition to the posttraining enhancements observed in Experiment 1 led us to a follow-up experiment to further examine the contribution of audition during training. We reasoned that if the posttraining improvement in Experiment 1 resulted from the presence of auditory information, then training with a visual-only moving stimulus would not be sufficient to yield this enhancement in posttraining performance when compared with the multisensory case of Experiment 1 (cf. Barakat et al., 2015). If, however, visual motion can mediate the rhythmic information needed for increasing discrimination accuracy, then the posttraining performance could be enhanced following training with visual-only moving stimuli. In Experiment 2, therefore, we kept the experimental structure and design of Experiment 1 with the sole difference of the training stimulation, which was now composed of visual-only rhythmic patterns. Specifically, we trained participants with the moving bar and static circles utilized in Experiment 1 in the absence of auditory tones (i.e., Vinan and Vstat group).

## Experiment 2

### Methods

#### Participants

Twenty-two new university students (18 female) aged between 19 and 20 years old (mean age = 19.5 years) took part in this experiment.

#### Apparatus, stimuli, design, and procedure

These were the same as for Experiment 1 with the sole exception that instead of being trained with multisensory rhythms, participants received a unisensory training with a moving bar (Vinan) or static circles (Vstat), where the visual stimulus was presented without the auditory tones at the onset and offset of each interval (see “Stimuli” for Experiment 1). The beat sequence was maintained (i.e., IBI = 800 ms). We used the moving bar as one of our training stimuli due to Grahn’s (2012) findings of visual moving stimuli mediating rhythmic information. We also used the static circles to further support our results from Experiment 1 by showing that the outcomes are, indeed, not driven by audio.

## Results and discussion

For all the analyses, Bonferroni-corrected t-tests (where *p* < .05 prior to correction) were used for all post hoc comparisons. When sphericity was violated, Greenhouse–Geisser correction was applied. The alpha level was set to 0.05 and the confidence interval to 95%.

### Training data

**Fig. 4:**
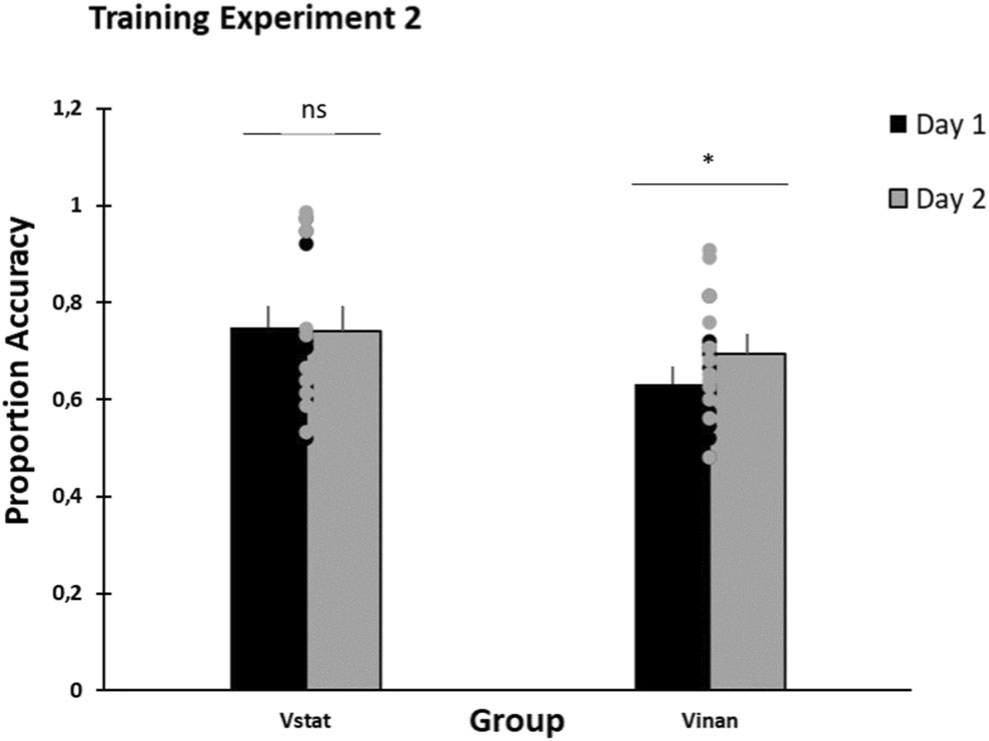
Mean discrimination accuracy during the two training sessions for the static circles (Vstat) and the moving bars (Vinan) training groups. Significant differences between the groups and the training sessions (*p* < .001) are indicated by two asterisks. The error bars represent the standard error of the mean

A mixed ANOVA with Training Session (2 levels: Session 1 vs. Session 2) and Rhythm Pair (25 levels) as the within-participant factors, and Group (Vstat, Vinan) as the between-participants factor was conducted. A significant main effect of Group, *F*(1, 19) = 1.894, *p* < .001, η_p_^2^= .091, CI [*NA*, .350] was obtained, with the static group performing significantly better (*M* = .746) as compared with the moving-bar group (*M* = .663; see Fig. 4). A main effect of Rhythm Pair was also obtained, *F*(24, 456) = 1.889, *p* = .007, η_p_^2^ = .090, CI [.007, 094], with some rhythmic pairs being particularly difficult to discriminate (M_BA_ = .630, M_BB_ = .616, M_BC_ = .597, M_BD_ = .674, M_BE_ = .657, M_CB_ = .653, M_CC_ = .664). We also obtained an interaction between Group and Rhythm Pair, *F*(24, 456) = 1.921, *p* = .006, η_p_^2^ = .092, CI [.008, .096], with some rhythm pairs being more accurately discriminated by the static training group (i.e., AB, AE, CA, DA, DE, EA, EB) as compared with the bar training group. We also obtained an interaction between Group and Training Session, *F*(1, 19) = 6.186, *p* = .022, η_p_^2^ = .246, CI [.002, .499]). The interactions between Training Session and Rhythm, *F*(24, 456) = 1.234, *p* = .206, η_p_^2^ = .061, and Training Session, Group, and Rhythm Pair, *F*(24, 456) = .809, *p* = .727, η_p_^2^ = .041, did not reach significance.

### Pre- and posttraining data

**Fig. 5:**
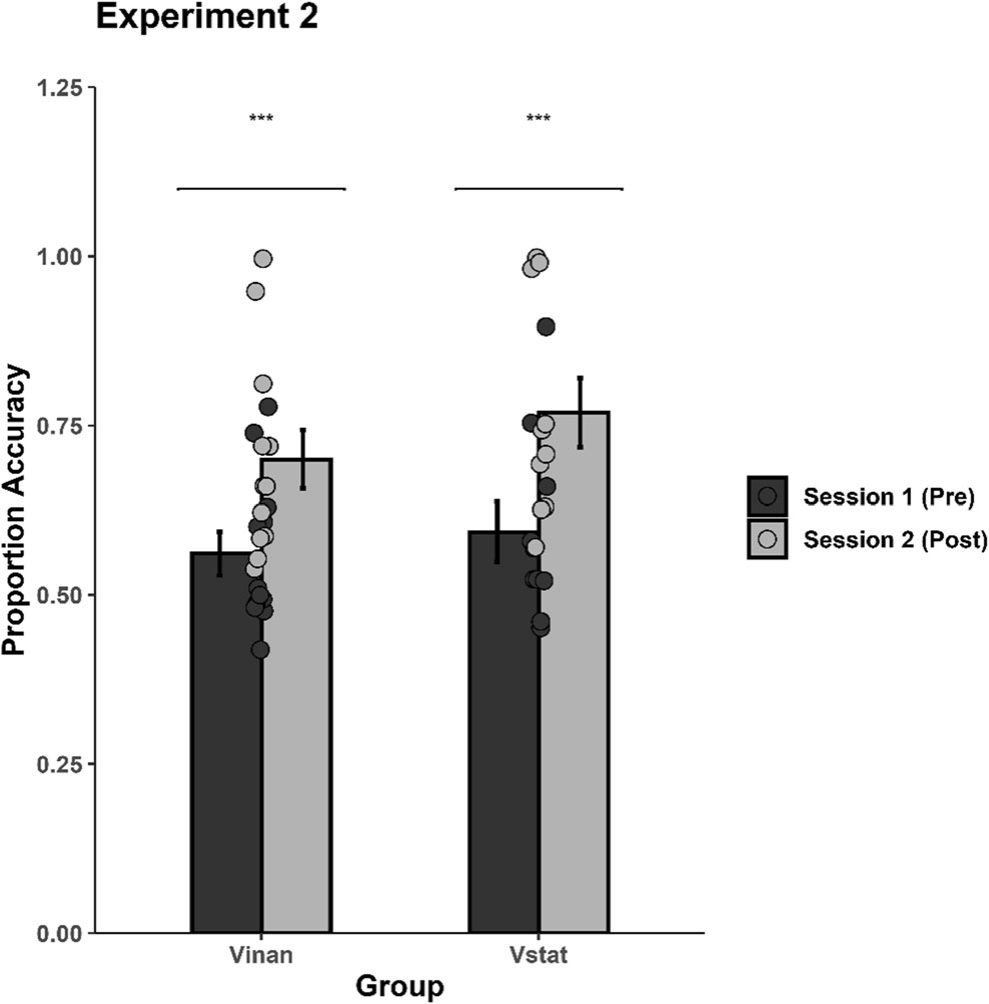
Mean discrimination accuracy during the pre- and posttraining sessions for the static circles (Vstat) and the moving bars (Vinan) training groups. Significant differences between the pre- and posttest sessions (*p* < .001) are indicated by two asterisks. The error bars represent the standard error of the mean

A mixed ANOVA with Session (2 levels: Pretraining vs. Posttraining) and Rhythm Pair (25 levels) as within-participant factors, and Group (2 levels: Vstat, Vinan) as the between-participants factor was conducted. A significant main effect of Session was obtained, *F*(1, 19) = 31.439, *p* < .001, η_p_^2^ = .623, CI [.289, .762], with both groups exhibiting a significant improvement in posttraining performance (*M* = .718) as compared with the pretraining (*M* = .691; see Fig. 5). Thus, despite the absence of the auditory tone stimulation in Experiment 2, training with visual-only moving stimuli continued to enhance posttraining performance in a task where the rhythms consisted of static visual stimuli.

We also obtained a significant interaction between Session and Rhythm Pair, *F*(24, 456) = 4.186, *p* < .001, η_p_^2^ = .181, CI [.081, 200], with 16 out of the 25 rhythm pairs being significantly more accurately discriminated during the posttraining as compared with the pretraining (i.e., AB, AD, BA, BC, BD, BE, CA, CB, CE, DA, DB, DC, EA, EB, EC, ED). The main effect of Group, *F*(1, 19) = 1.370, *p* = .256, η_p_^2^ = .067, Rhythm, *F*(24, 456) = 1.467, *p* = .061, η_p_^2^ = .073, and the interactions between Session and Group, *F*(1, 19) = .284, *p* = .60, η_p_^2^ = .015, Rhythm Pair and Group, *F*(24, 456) = 1.467, *p* = .072, η_p_^2^ = .072, and Session, Rhythm Pair, and Group, *F*(24, 456) = 1.267, *p* = .180, η_p_^2^ = .063, did not reach significance.

Overall, the results of Experiment 2 demonstrated that visual-only training with either a static or moving stimulus can enhance rhythm perception even in the absence of auditory rhythmic stimulation. In particular, training with a visual-only moving (i.e., a moving bar) or static stimulus (i.e., static circles) improved the participants’ processing and discrimination ability of metric simple visual rhythms consisting of static stimuli. More importantly, we did not find any enhancement differences between visual static and visual moving training, suggesting that both visual stimuli are sufficient to improve discrimination accuracy of the two integer-ratio visual rhythms we used in the pre- and posttraining sessions. One potential explanation for the enhancement after the visual moving training could also be that the intervals between targets could have helped participants to accurately predict their location (Pfeuffer et al., 2020; Wagener & Hoffmann, 2010). Moreover, participants are able of learning such complex spatiotemporal patterns and this process most likely is implicit (Kirkham et al., 2007). However, until now, training with nonoptimal rhythms has not been found sufficient to improve rhythm discrimination (Barakat et al., 2015; Zerr et al., 2019). Thus, it is possible that the similarity of visual static stimuli with the main task stimuli influenced the participants’ performance.

To compare task performance after unisensory and multisensory training, we performed a combined analysis of the data from Experiment 1 (AVstat, AVinan) and Experiment 2 (Vstat, Vinan). For the training data, we used a mixed ANOVA with Training Session (2 levels: Session 1 vs. Session 2) and Rhythm Pair (25 levels) as within-participant factors, and Group (four levels: AVstat, AVinan, Vstat, Vinan) as the between-participants factor. We obtained a main effect of Training Session, *F*(1, 53) = 11.751, *p* = .001, η_p_^2^ = .181, CI [.032, .353] with the participants’ performance being significantly better in Session 2 (*M* = .800) than in Session 1 (*M* = 772). We also obtained a main effect of Rhythm Pair, *F*(12.876, 682.429) = 6.527, *p* < .001, η_p_^2^ = .110, CI [.055, .139], with some rhythms having systematically lower accuracy (M_CB_ = .681, M_CD_ = .688, M_DD_ = .679) compared with others (M_AD_ = .871, M_AE_ = .856, M_EB_ = .880, M_EC_ = .858). A significant main effect of Training group was observed, *F*(1, 53) = 14.179, *p* < .001, η_p_^2^ = .445, CI [.048, .382], with the groups AVstat (*M* = .916) and AVinan (*M* = .819) performing better than Vstat (*M* = .746) and Vinan (*M* = .663). The interaction between Rhythm pair and Training group was also found significant, *F*(38.628, 682.429) = 2.201, *p* < .001, η_p_^2^ = .111, CI [.025, .105], with the training groups discriminating more accurately specific rhythms. The interactions between Training Session and Group, *F*(3, 53) = 2.461, *p* = .073, η_p_^2^ = .122, Training Session and Rhythm pair, *F*(1.809, 59.119) = 1.622, *p* = .063, η_p_^2^ = .030, and Training Session, Rhythm Pair, and Group, *F*(3.869, 59.119) = 44.604, *p* = .228, η_p_^2^ = .061, were not found significant.

For the pre- and posttraining data, we conducted a mixed ANOVA with Session (2 levels: Pretraining vs. Posttraining) and Rhythm Pair (25 levels) as within-participant factors, and Group (4 levels: AVstat, AVinan, Vstat, Vinan) as the between-participants factor. A significant main effect of Session was obtained, *F*(1, 54) = 77.224, *p* < .001, η_p_^2^ = .590, CI [.407, .696], with all groups performing better posttraining (*M* = .745) than pretraining (*M* = .613). A main effect of Rhythm Pair was also obtained, *F*(10.571, 77.682) = 6.527, *p* < .001, η_p_^2^ = .120, CI [.230, .535], with some rhythm pairs being particularly difficult to discriminate (M_BC_ = .579, M_BB_ = .616, M_CB_ = .522, M_DE_ = .585). Additionally, an interaction between Session and Rhythm Pair, *F*(7.960, 66.459) = 6.468, *p* < .001, η_p_^2^ = .107, CI [.189, .520], and Rhythm Pair and Training Group, *F*(7.221, 77.682) = 1.673, *p* = .006, η_p_^2^ = .085, CI [*NA*, .207], was also obtained. The main effect of Training group and the interactions between Session and Training Group, *F*(3, 54) = 1.198, *p* = .319, η_p_^2^ = .062, and Session, Rhythm Pair, and Training Group, *F*(4.806, 66.459) = 1.302, *p* = .100, η_p_^2^ = .067, did not reach significance. The results of this combined analysis further support our previous findings that unisensory training with moving or static stimuli can indeed improve rhythm discrimination accuracy.

So far, it is not clear whether the results obtained in the two experiments described were due to perceptual learning or due to the mere exposure to the experimental conditions. Perceptual learning can be defined as the performance enhancement on a task, emerging from prior perceptual experience (Watanabe & Sasaki, 2015). This enhanced performance comes, commonly, because of a training with feedback period (Dosher & Lu, 2017; Hammer et al., 2015). However, it is possible that improvement can also occur after a period of mere exposure to numerous stimulus features (Liu et al., 2010; Watanabe et al., 2001). To address this potential “mere exposure” confound, we conducted a control experiment to further examine whether an effect of session (pre- vs. posttraining) will be obtained in the absence of feedback during training. If this effect is present in the control experiment, the results obtained in Experiment 1 and Experiment 2 may simply reflect mere exposure effects rather than learning processes. However, if no posttraining enhancements occur in the absence of feedback, our effects can be attributed to perceptual learning processes.

**Fig. 6:**
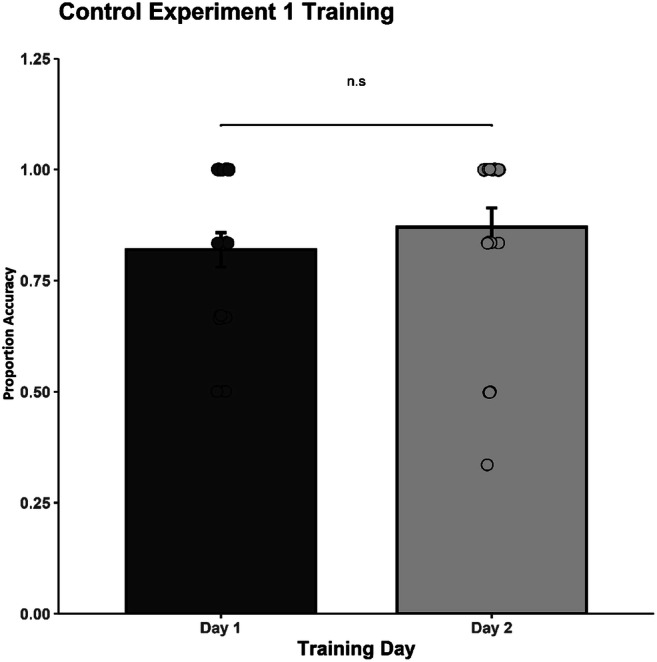
Mean discrimination accuracy during the two training sessions for the control group. Significant differences between the training sessions (*p* < .001) are indicated by two asterisks. The error bars represent the standard error of the mean

Twenty-three university students (21 females) aged between 20 and 37 years old (mean age = 22.5 years) took part in this control experiment. We kept stimuli, design, and procedure identical to Experiment 1 with only two exceptions: we removed feedback during the training sessions, and we only utilized the AVinan condition in order to reduce experimentation time. The training data were analyzed using a repeated-measures ANOVA between Session (two levels: Session 1, Session 2) and Rhythm Pair (25 levels). The alpha level was set to 0.05 and the confidence interval to 95%. The analyses showed no statistically significant main effect of Session, *F*(1, 22) = 2.49, p = .129, η_p_^2^ = .10, with performance during Session 2 (*M* = .769) remaining at the same level with Session 1 (*M* = .746; see Fig. 6). A main effect of Rhythm Pair was obtained, *F*(24, 528) = 7.06, *p* < .001, η_p_^2^ = .24, CI [.159, .260], with some rhythmic pairs being particularly difficult to discriminate (i.e., AC, BC, CD, DC, DE, ED). The interaction between Training Session and Rhythm Pair, *F*(24, 528) = 0.96, *p* = .524, η_p_^2^ = .04, did not reach significance.

**Fig. 7:**
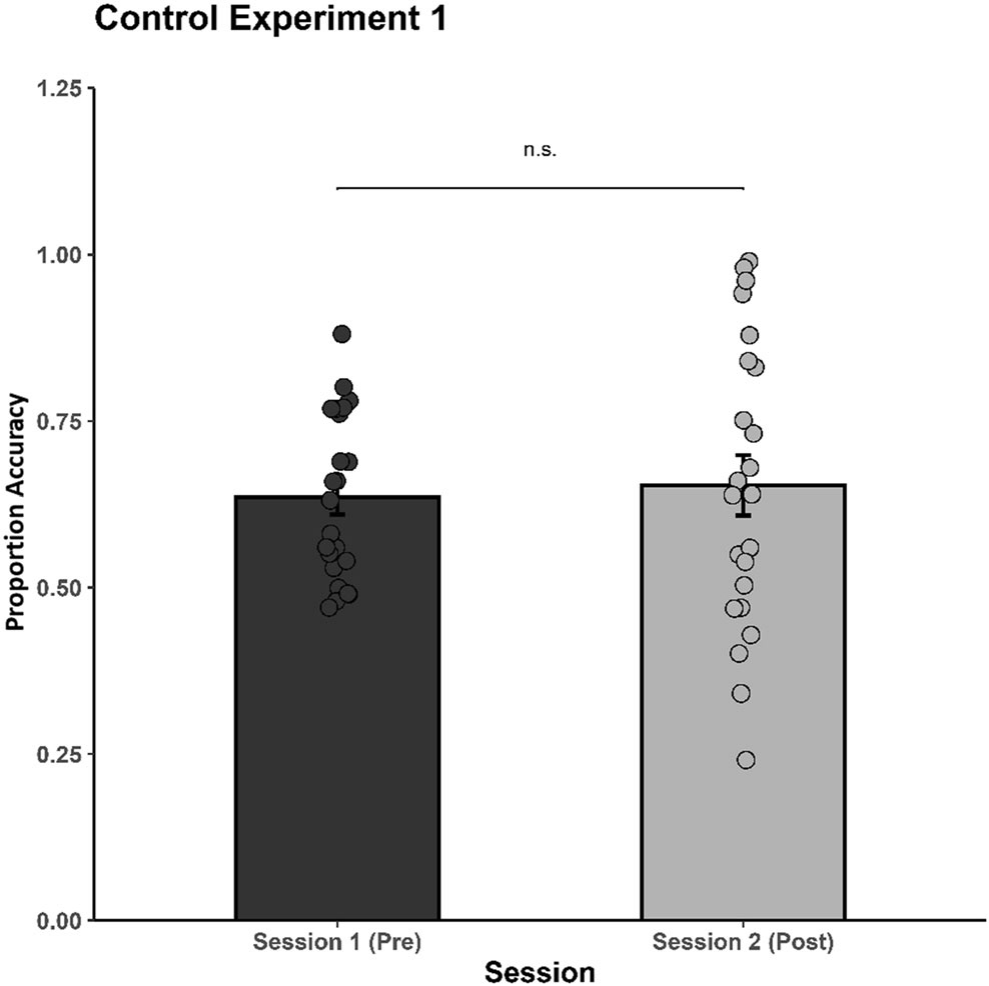
Mean discrimination accuracy during the pre- and posttraining sessions for the control group. Significant differences between the pre- and posttest sessions (*p* < .001) are indicated by two asterisks. The error bars represent the standard error of the mean

**Fig. 8:**
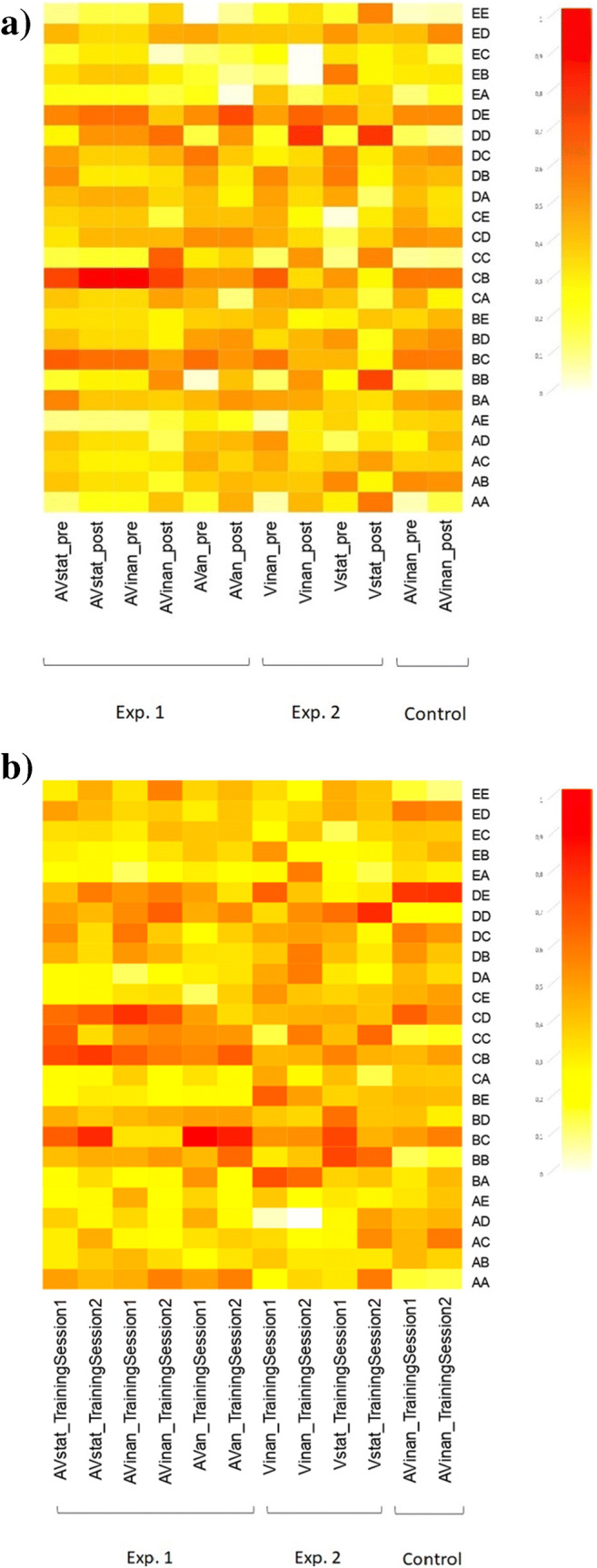
The above heatmaps depict (**a**) the mean accuracies of the 25 rhythm pairs used in the pre- and posttraining sessions, grouped by training group (AVstat, AVinan, Avan, Vinan, Vstat) and session (Pre-, Posttraining) for Experiments 1 and 2 and the Control experiment, (**b**) the mean accuracies of the 25 rhythm pairs used in each training group, grouped by training group (AVstat, AVinan, Avan, Vinan, Vstat) and session (Training Session 1, Training Session 2) for Experiments 1 and 2 and the Control experiment. The lower mean accuracies are associated with lighter colorings (i.e., yellow) while the higher mean accuracies are associated with darker colorings (i.e., red). (Colour figure online)

The pre- and posttraining data were analyzed by using a repeated-measures ANOVA between Session (two levels: Pretraining vs. Posttraining) and Rhythm Pair (25 levels). The main effect of Session did not reach significance, *F*(1, 22) = 0.52, *p* = .480, η_p_^2^ = .02 (see Fig. 7), so in the absence of feedback during training we did not obtain a significant improvement in performance. However, a main effect of Rhythm Pair was revealed, *F*(9.14, 201.12) = 10.40, *p* < .001, η_p_^2^ = .32, CI [.207, .372], with some rhythm pairs having systematically lower accuracy scores (i.e., AB, BA, BC, BD, CB, CD, DB, DC, DE, ED; see also Fig. 8 for a summation on rhythms mean accuracy). The interaction between Rhythm Pair and Session, *F*(10.33, 227.24) = 1.26, *p* = .253, η_p_^2^ = .05, did not reach significance. Overall, the results suggest that the findings we obtained in Experiments 1 and 2 can be attributed to perceptual learning processes, since the same design, but without feedback (supported as essential to learning; Goldhacker et al., 2013), did not yield significant results.

## General discussion

In the present study, we used a rhythm perceptual learning paradigm, where we manipulated the type of the visual stimulus (i.e., moving vs. static and animate vs. inanimate; Experiment 1) and the training modality (Experiment 2) so as to investigate the potential of posttraining enhancement of visual rhythm processing. Our results showed that visual rhythm perception can be enhanced when using both moving and static and/or animate and inanimate stimuli in training, when the rhythmic information is mediated by audiovisual (Experiment 1) or visual only stimuli (Experiment 2), but only if trial-by-trial feedback is provided during training (Control experiment).

The main aim of Experiment 1 was to assess the effects of stimulus attributes during perceptual training (e.g., visual movement or animacy) on the discrimination of subsequently presented rhythms consisting of nonoptimal static stimuli. Previous studies employing discrimination tasks have reported that compared with visual rhythms consisting of static stimuli, exposure to visual stimuli with spatiotemporal information (e.g., visual movement) may increase the temporal reliability of visual rhythm encoding (Gan et al., 2015; Grahn, 2012; Hove et al., 2013), and, thus, facilitate rhythm processing (Grahn, 2012; Hove et al., 2013). Given that no studies have tested the effects of visual movement on rhythm perceptual learning, we reasoned that the latter findings may explain why perceptual learning effects are not observed following training with visual-only, nonoptimal, static rhythms (Barakat et al., 2015). To address this possibility, we manipulated the visual component of the multisensory rhythms during training. However, contrary to our predictions, the results obtained in Experiment 1 showed that all three different forms of training led to a significantly better posttraining performance, irrespective of the type of the visual stimulation presented. Consistent with previous evidence reporting an auditory advantage in rhythm processing (Collier & Logan, 2000; Patel et al., 2005) and auditory-driven training effects in perceptual learning tasks (Barakat et al., 2015), one possible explanation of our findings is that the similar performance between the different training groups could be driven by the multisensory benefit during training, and, in particular, from the contribution of audition to the multisensory stimulus presented, in agreement with the optimal integration hypothesis (Ernst & Banks, 2002).

A secondary aim of Experiment 1 was to investigate whether animate or inanimate moving stimuli exert differential influences on subsequent visual rhythm processing. However, contrary to previous findings supporting that biological motion affects time estimates (Blake & Shiffrar, 2007; Carrozzo et al., 2010; Lacquaniti et al., 2014; Mendonça et al., 2011; Orgs et al., 2011), facilitates temporal prediction of actions as compared with inanimate moving stimuli (Stadler et al., 2012), and improves synchronization and rhythm discrimination accuracy (Su, 2014a, 2014b, 2016; Su & Salazar-López, 2016; Wöllner et al., 2012), we did not observe any animacy-related enhancements in Experiment 1. This could potentially be attributed to the stimulus design adopted (i.e., Su, 2014b). Specifically, as in Su’s study, the PLF’s movement in Experiment 1 had a fixed timing (i.e., the repetitive movement lasted 500 ms in Su’s study and 800 ms in our study), thus not mediating the duration of the accompanying auditory rhythm, which consisted of intervals of different durations (i.e., 250, 500, 750, or 1000 ms and 400 or 800 ms in Su’s and our study, respectively). This may have minimized the potential of observing an animacy-driven benefit (AVan) when comparing the performance between the different multisensory training groups. The absence of animacy-related enhancements in our study raises the possibility that the beneficial impact of biological motion reported by Su (2014b) could be due to modality differences that resulted from comparing performance between multisensory (auditory tones and PLF) and auditory-only rhythms. We, therefore, suggest that the enhancements reported by Su in the multisensory as compared with the auditory-only trials were not due to the beneficial impact of biological motion on rhythm processing but instead due to the behavioral benefits associated with multisensory as compared with unisensory stimulation (e.g., Huang et al., 2012; Stein & Stanford, 2008). Future studies need to account for the temporal aspects of auditory and visual rhythms that are mediated by biological motion to gain a better understanding of the potential animacy-related enhancements in rhythm processing.

In Experiment 2, we eliminated the potential effects of auditory dominance in Experiment 1 since posttraining enhancement was also obtained despite the absence of auditory information. While, indeed, visual-only moving stimuli have been found to improve rhythm perception (Grahn, 2012; Hove et al., 2013; Repp & Su, 2013), our study extends that body of literature by being the first to investigate whether the processing of two integer-ratio metric simple visual rhythms consisting of untrained static stimuli can be enhanced after training with rhythms containing motion information. Contrary to the findings reported by Zerr et al. (2019) and Barakat et al. (2015), we found that visual-only training can be as efficient as multisensory training in improving posttraining discrimination performance. The absence of significant posttraining enhancements for the visual-only training group in the above-mentioned studies might, thus, simply reflect the inefficiency of training with visual-only static stimuli in yielding learning effects, since the visual system rarely processes temporal information that lacks a spatial translation (Hove et al., 2013). Overall, while exposure to auditory stimulation have been found to facilitate subsequent visual rhythm processing (Barakat et al., 2015; Collier & Logan, 2000; Grahn et al., 2011; McAuley & Henry, 2010), our results are the first to extend these findings by showing that discrimination of two integer-ratio visual rhythms consisting of static stimuli can be facilitated by prior exposure to both audiovisual (Experiment 1; cf. Barakat et al., 2015; Zerr et al., 2019) and visual (Experiment 2) stimuli. The ability to predict the timing and the allocation pattern of an event can enhance information processing and this can be achieved when this event appears rhythmically (i.e., speech, music, biological motion; Breska & Deouell, 2017). Johndro et al. (2019) further investigated auditory rhythms and found that their temporal characteristics are able of directing attention and, moreover, enhancing the encoding of visual stimuli into memory. Our results extend previous work by showing that the spatiotemporal characteristics of visual dynamic rhythms enhance the processing of static visual rhythms but only in the presence of feedback (Control experiment). The use of such predictive stimuli during training trials failed to enhance posttraining performance in the absence of feedback.

An alternative explanation of our findings would be that they may reflect enhancements due to mere exposure to the rhythms during training rather than learning processes. Given that feedback is indeed important for learning to occur (Dosher & Lu, 2017), we addressed this possibility by performing a control experiment without feedback during the training sessions. Through this control experiment, we showed that the posttraining enhancements obtained depend on the presence of feedback during training. These results are also in line with evidence showing that training with trial-by-trial feedback enhanced temporal acuity for audiovisual stimuli unlike simple exposure to the stimuli (De Niear et al., 2017). Most perceptual learning studies use trial-by-trial feedback as it is correlated with performance improvement, yet learning can be the result of block feedback as well as no feedback at all (Liu et al., 2014). Moreover, while feedback was mainly seen as a way to make perceptual learning easier rather than produce it, Choi and Watanabe (2012) support that feedback can induce learning in an orientation discrimination task by increasing participants’ sensitivity even for trials where the actual stimuli were replaced by noise.

In the experiments described in this paper, we utilized metrical and low complexity rhythms that consisted of only two types of intervals (i.e., two-integer-ratio metric simple rhythms). This might explain the high accuracy scores we observed even during pretraining. It remains unknown, whether this training-driven enhancement would be evident in more complex rhythms (e.g., three- or four-integer-ratio rhythms). Although feedback may be considered as an important factor of perceptual learning (Powers et al., 2009; Seitz et al., 2006), training task difficulty seems to affect performance and maybe interact with feedback. Indeed, De Niear et al. (2016) suggested that harder training procedures may lead to a noticeable performance increment, while Goldhacker et al. (2013) claimed that feedback might be helpful for easier tasks, but it can prevent learning when it comes to more challenging ones. On the other hand, Gabay et al. (2017) claimed that an overall highly intense training procedure may not be as productive as an easier one, while Sürig et al. (2018) suggested that adapting training task difficulty to each participant’s abilities can lead to the optimal learning outcomes. Future experimentation may allow a clearer picture in terms of role of task difficulty in training and perceptual learning.

Increasing task difficult by increasing the number of intervals would potentially result in increased memory load, thereby rendering it unlikely to efficiently store and process the rhythmic patterns presented, which could, in turn, affect the transfer of learning (Teki & Griffiths, 2014). This is in line with the Scalar Expectancy Theory (SET), an internal clock model presented by Gibbon (1977). The SET model shares some common elements with previous internal clock models (cf. Creelman, 1962; Treisman, 1963) like the pacemaker, the counter/accumulator, and the decision process, but with the addition of a mechanism consisting of two memory stores, the working memory (short term) and the reference memory (long term) store. When timing a stimulus, pulses are produced by the pacemaker, which are then collected at the level of the accumulator. Those pulses are stored in working memory and are compared with those that were already stored in the reference memory. Then, a decision-making process allows for the time estimation requested by the participant. What is interesting about this system is that it can be initiated, paused, and reset with the aim of providing time estimations for individual or multiple events that take place at the same time (Allman et al., 2014). Moreover, while the mean duration of a time interval increases, the standard deviation of the duration estimate increases as well, thus attributing the naming scalar to this model (Rhodes, 2018). Indeed, considering the SET beyond the context of a single interval, it has been hypothesized that the reference memory gets overloaded with increasing number of intervals, thereby resulting in worse memory performance (Teki & Griffiths, 2014). This is in line with evidence supporting that rhythm discrimination tasks require working memory resources so as to compare the standard rhythms to the comparison stimuli (Leow & Grahn, 2014), while studies have also shown that rhythms of more integer ratios are less efficiently processed as compared with two integer-ratio rhythms (i.e., as those used in Experiment 1; Drake, 1993). This is probably why multisensory training can yield enhanced posttraining performance in a visual-only rhythm discrimination task with two integer-ratio rhythms (cf. Experiment 1; Barakat et al., 2015), while four integer-ratio visual rhythms of static stimuli cannot be accurately discriminated (Collier & Logan, 2000). The effects of integer-ratio on rhythm processing are further supported by neuroimaging data showing that when compared with simple isochronous rhythmic sequences, the processing of four integer-ratio metric rhythms results in increased activation in the superior prefrontal cortex, an area that has been suggested to be responsible for the memory representation of more complex rhythm sequences (Bengtsson et al., 2009). Taken together, these findings suggest that studies that test one or two different interval lengths, as the ones used in this study, cannot necessarily be generalized to timing of four different interval lengths (Grahn, 2012). Future work is needed to investigate the modulatory effects of integer-ratio on rhythm perceptual learning, by testing whether learning effects can be obtained across different levels of rhythm complexity (e.g., rhythms consisting of two or more integer ratios).

In conclusion, utilizing a perceptual learning paradigm, we showed that the processing of nonoptimal visual rhythms benefits from training with multisensory and visual moving or static stimuli. Moreover, we showed that these benefits are unlikely to be the result of a mere effect of exposure since no enhancements were found in the absence of feedback. However, given that we used low complexity metric simple rhythms, we suggest that the role of task difficulty in rhythm perceptual learning should be further investigated. Future work should also aim to highlight the rhythmic structure of visual sequences by using more naturalistic and complex body movements (e.g., dancing) that might be more efficient in communicating the rhythmic information through different body parts so as to further optimize visual rhythm perception.
